# Feasibility of tracking invasive *Escherichia coli* disease among older adults in a community setting: A prospective observational study

**DOI:** 10.1007/s10096-023-04738-y

**Published:** 2024-01-18

**Authors:** Joachim Doua, Miquel Ekkelenkamp, Theo Verheij, Oscar Go, Stephen Ruhmel, Kimberly Leathers, Bart Spiessens, Sanne van Rooij, Vance G. Fowler, Jeroen Geurtsen, Rowena Dolor, Michal Sarnecki, Ranee Chatterjee, Jan Poolman, Marc Bonten, on behalf COMBACTE-NET consortium/EXPECT study group

**Affiliations:** 1 Janssen Research & Development, Beerse, Belgium; 2 Department of Medical Microbiology, University Medical Center Utrecht, Utrecht University, Utrecht, the Netherlands; 3 Julius Center for Health Sciences and Primary Care, University Medical Center, Utrecht University, Utrecht, the Netherlands; 4 Janssen Research & Development, Raritan, NJ USA; 5 Janssen Research & Development, Titusville, NJ USA; 6 Duke University School of Medicine, Durham, NC USA; 7 Department of Medicine, Duke University Medical Center Durham, Durham, NC USA; 8 Duke Clinical Research Institute, Durham, NC USA; 9 Bacterial Vaccines Discovery and Early Development, Janssen Vaccines & Prevention B.V, Leiden, the Netherlands; 10 Janssen Vaccines, Branch of Cilag GmbH International, Bern, Switzerland; 11 IQVIA, Research Triangle Park, Durham, NC USA

**Keywords:** Invasive *Escherichia coli* disease, Extraintestinal pathogenic *Escherichia coli*, *E. coli*, Bacterial infection, Vaccine, Urinary tract infection

## Abstract

**Purpose:**

Invasive *Escherichia coli* disease (IED) encompasses a diverse range of sterile site infections. This study evaluated the feasibility of capturing IED among community-dwelling older adults to inform the implementation of a phase 3 efficacy trial of a novel vaccine against IED (NCT04899336).

**Methods:**

EXPECT-1 (NCT04087681) was a prospective, multinational, observational study conducted in medically stable participants aged ≥ 60 years. At least 50% of participants were selected based on a history of urinary tract infection (UTI) in the previous 10 years. The main outcomes were the incidence of IED and the number of hospitalisations reported by the site vs participant. The length of follow-up was 12 months. In a US-based substudy, a smartphone-based geofencing was evaluated to track hospital entries.

**Results:**

In total, 4470 participants were enrolled (median age, 70.0 years); 59.5% (2657/4469) of participants had a history of UTI in the previous 10 years. Four IED events were captured through deployment of different tracking methods: a self-report, a general practitioner (GP) report, and a follow-up call. The incidence rate of IED was 98.6 events per 100,000 person-years. The number of reported hospitalisations was 2529/4470 (56.6%) by the site and 2177/4470 (48.7%) by participants; 13.8% of hospitalisations would have been missed if utilising only site reports. Geofencing detected 72 hospital entries.

**Conclusion:**

Deployment of multiple tracking methods can optimise detection of IED among community-dwelling older adults. Older adults with a history of UTI could be feasibly targeted for a phase 3 vaccine efficacy trial through a network of GPs.

**Supplementary Information:**

The online version contains supplementary material available at 10.1007/s10096-023-04738-y.

## Introduction

Extraintestinal pathogenic *Escherichia coli* (ExPEC) can infect sterile sites outside the gastrointestinal tract and cause a broad range of invasive diseases [1]. *E. coli* is the most common pathogen causing bloodstream infections (BSIs) in population-based studies [2] and is the most frequently isolated pathogen in patients with sepsis in the United States [3, 4]. Invasive *E. coli* disease, also known as invasive ExPEC disease (IED), can be defined as an acute illness consistent with systemic bacterial infection microbiologically confirmed by a positive *E. coli* culture from a normally sterile body site (including blood) or from urine in patients with urosepsis and no other identifiable source of infection [5].

On a global scale, *E. coli* was identified as the number one pathogen linked to global deaths attributable to and associated with antimicrobial resistance [6]. Older adults might have an increased risk for developing IED. Estimates from a recent systematic literature review indicate a substantially increased incidence of *E. coli* bacteraemia among adults ≥ 60 years of age relative to the population average with 110, 154, and 319 episodes per 100,000 person-years among those aged 60-to-69 years old, 70-to-79 years old, and 80 years and older, respectively. [7]. Furthermore, the highest level of *E. coli* non-susceptibility to antibiotics in BSIs was reported among adults aged ≥ 65 years in a study from England [8].

A novel vaccine to prevent IED in older adults is currently being evaluated in a phase 3, double-blind, placebo-controlled, randomised trial [9]. In this trial, community-dwelling older adults are enrolled, and the primary endpoint, IED, needs to be captured in a hospital setting. The performance of such a trial critically depends on the use of a sufficiently large sample size to enable efficient endpoint catchment. A placebo-controlled and double-blind design guarantees study validity; however, not capturing all endpoints may render a trial underpowered when using a fixed follow-up duration or prolong the follow-up period when using an endpoint-based approach. Among the elderly, blunted or non-specific presenting symptoms of invasive disease [10] can complicate IED catchment. Additionally, IED catchment in hospitals may be limited by transfer of participants with acute disease to sites other than those participating in the trial. One data collection solution involves utilising a combination of conventional data collection approaches (eg, medical record review and participant self-report) and novel digital technologies. For example, a smartphone-based geofencing application leverages Wi-Fi and global positioning system capabilities of personal cellular devices to electronically detect hospital visits and ascertain hospitalisations [11].

EXPECT-1 was a prospective, multinational, observational study assessing the feasibility of capturing IED events and hospitalisations among community-dwelling adults aged ≥ 60 years through deployment of study site reports and participants’ self-reports. A US-based substudy evaluated the usefulness of geofencing to track hospital entries. EXPECT-1 was conducted as part of the Combatting Bacterial Resistance in Europe–Networks (COMBACTE–NET) project [12]. The findings from this study aimed to inform the implementation of an ongoing phase 3 trial of a vaccine against IED in older adults with a history of urinary tract infection (UTI) in the past 2 years [9].

## Methods

### Design, setting, and recruitment

EXPECT-1 was a prospective, multicentre, observational study conducted across networks of 8 hospital sites each servicing patients from primary care (PC) centres in Canada, France, Germany, Italy, Spain, United States, United Kingdom, and Japan. The study was initiated on 20 September 2019 and was completed on 31 May 2021 (ie, the date of the last participant’s last visit and completion of follow-up). Each participating country had a local network of general practitioner (GP) and PC centres encompassing a population of approximately 40,000 individuals and a single local hospital where patients with suspected IED were referred. Recruitment was performed through electronic health record database screening by GP/PC centres and supported by the local research team (LRT; collectively referred to as “the site”).

Before enrolment, each participant provided written or electronic informed consent authorising access to their medical records in compliance with applicable data privacy laws. The study was conducted in compliance with the Declaration of Helsinki and Good Clinical Practices. Due to the COVID-19 pandemic, enrolment and follow-up visits at some sites were transitioned from in-person to virtual.

### Population

Eligibility criteria included the following: willingness to provide written informed consent, age ≥ 60 years, availability for contact with the site for the duration of the study, ambulatory and living in the community (or with minimal assistance in an assisted-living or long-term care residential facility) and deemed medically stable by the site. Exclusion criteria were serious chronic disorder, history of malignancy within 5 years prior to screening, major psychiatric illness (and/or substance abuse or substance use disorder), and the likelihood of non-adherence (Supplementary Table 1). The sites were instructed to enrol ≥ 50% of participants with a history of ≥ 1 UTI in the previous 10 years.

### Endpoints

The main endpoints were the incidence of IED; the number of hospitalisations and the number of participants with any medical encounter, as reported by the site vs participants, in all participants and in patients with IED. For the purposes of this analysis, only participants that developed an initial IED during the 1-year follow-up and were hospitalized are reported. Among patients with IED, additional endpoints were evaluated: clinical features, diagnostic methods, treatment, and IED risk-related medical history including the rate of complicated and uncomplicated UTI, recurrent UTI, cardiovascular disease, gastrointestinal disease, stroke, diabetes mellitus, urolithiasis, and urological intervention (Supplementary Table 2).

### Data sources and endpoint measurement

Baseline demographic and medical history data on all participants were collected by the site on the day of enrolment (day 1) and entered into the electronic case report form. Medical history evaluation included a history of UTI, a history of IED, chronic disorders, the use of long-term medication at the time of enrolment or recruitment into the study, and collection of standard criteria for IED vaccination based on the criteria for the phase 3 vaccine efficacy study (NCT04899336). Among participants with IED, medical history included conditions with increased risk for IED, other relevant coexisting medical conditions, and any relevant treatment received in the 3 months prior to IED onset, ie, immunosuppressive therapy (steroids, anti-cancer chemotherapy, radiation therapy, or cytotoxic drugs) and antibiotics (prophylactic or therapeutic).

Follow-up data were collected through 12 months at study sites through medical file review, 4-month phone calls, and a study participation card, as well as through participants’ self-reports. Follow-up visits were conducted by phone at 4, 8, and 12 months after enrolment to capture information about development of IED and medical resource utilisation (MRU; i.e., medical encounters and hospital admissions). In addition, participants were provided with a study participation card that could have been used by a relative or a treating physician to report a hospital admission or IED to the site if the participant was unable to do so. In parallel, any referral to the hospital or outpatient clinic during follow-up was tracked by the site. Participants were also asked to inform the site in case of a hospital admission. To collect new information about IED and MRU, participants’ medical files were checked at the end of the study. Medical encounters recorded into the electronic case report form could have started before the 12-month follow-up period. When reported, IED events were assessed through medical record review. Diagnostic criteria for IED were based on a combination of medical judgement informed by physicians’ notes and *International Classification of Diseases* codes (Supplementary Table 3). IED was classified as bacteraemic when *E. coli* was cultured from blood; otherwise, IED was considered non-bacteraemic.

Among identified IED events, the data were collected on day 1 (date of diagnosis) and day 28 after IED diagnosis. Data collection on day 1 included data on IED signs and symptoms, laboratory data, treatment for IED, concomitant medication, and MRU (hospital admissions and medical encounters). On day 28, additional clinical and laboratory data were collected, as well as data on IED treatment, MRU, and recovery and discharge from the hospital. All study-related data and samples were collected as part of standard procedures and entered into the electronic case report form.

### Geofencing

A geofencing substudy was conducted in a subset of US-based participants. During the enrolment visit, participants were asked whether they would be willing to take part in the geofencing substudy. After expressing interest, participants completed a separate consent form, and the study coordinator provided instructions for downloading the application on their personal cellular device.

The geofencing application detects a participant’s crossing into a pre-programmed healthcare facility location. Subsequent to detecting crossing, the geofencing application delivers a questionnaire to a participant’s smartphone to determine the reason for the visit. Depending on the answer, the site may be alerted within minutes of the participant entering the location. In case of suspected IED, the application alerts the study staff to triage potential cases.

### Statistical analyses

Data analysis was descriptive and no formal hypothesis testing was performed. The planned sample size was 6,000. Participant demographic and clinical characteristics were described by frequency distributions (categorical variables) and descriptive statistics (continuous variables).

The cumulative incidence was estimated by a ratio of the total number of IED events to the total number of participants in the full analysis set (FAS). The incidence rate was estimated by a ratio of total number of IED events to the total length of IED-free follow-up time (ie, the number of IED events per 100,000 person-years). A participant was included in the denominator regardless of subsequent study discontinuation or unknown IED status. The 95% CIs of cumulative incidence were based on the exact Clopper-Pearson interval; 95% CI of incidence rate were based on the exact Poisson interval.

## Results

### Baseline characteristics (FAS)

**Fig. 1:**
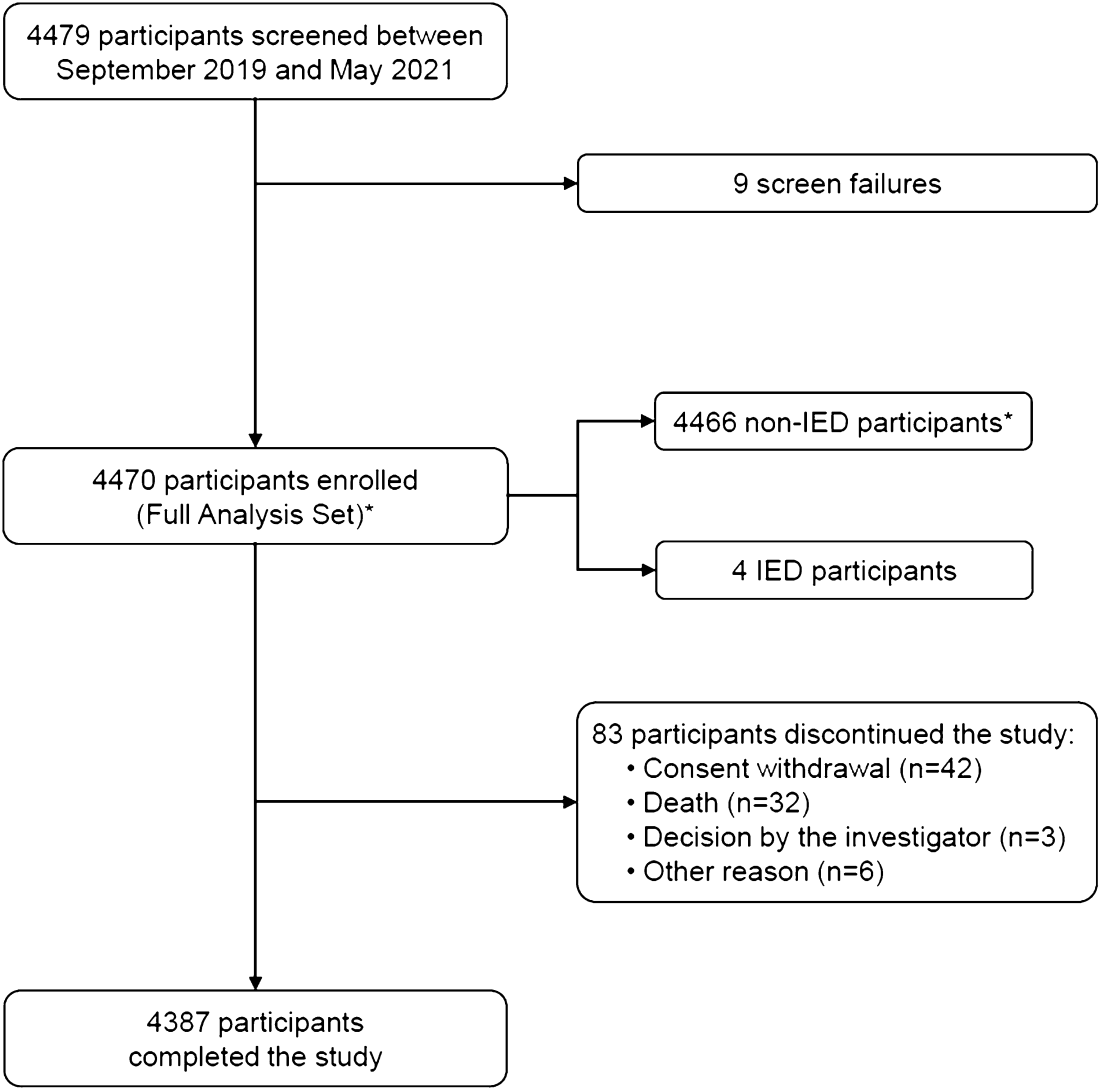
Study flow diagram, Footnote: *One participant was enrolled and withdrew consent on the same day. This subject was classified as non-IED. *IED* invasive *Escherichia coli* disease

Prior to the COVID-19 pandemic, the planned sample size of 6,000 was reduced to 5,500 participants. Due to the COVID-19 pandemic, the sample size was decreased to 4,500 participants. From 20 September 2019 through 31 May 2021, 4,479 participants were screened, and 4,470 were enrolled (FAS) (Fig. 1). FAS was the primary analysis set for all endpoint analyses and included participants fulfilling the eligibility criteria.

Baseline characteristics are summarised in Table 1 (median age, 70.0 years [range, 60–96]; 68.0% female). Among participants with a medical comorbidity (94.4%), comorbidities reported in ≥ 20% of participants were cardiovascular (64.9%), endocrine and metabolic (46.8%), musculoskeletal (43.4%), genitourinary (31.2%), and gastrointestinal disease (26.9%). In the previous 10 years, UTI was reported in 59.5% of participants, and IED in 7.2% of participants (Supplementary Table 4). Females comprised 77.5% of participants with a history of UTI and 54.1% of participants without a history of UTI (Supplementary Table 5). At the time of enrolment or recruitment into the study, 92.1% of participants (4117/4470) used medication; 6.4% (288/4470) used antibiotics. Through 12-month follow-up, 24.1% of participants (1077/4470) used antibiotics.

**Table 1:** Summary of key demographic and baseline characteristics (FAS)

	IED	All participants
FAS, n	4	4470
Age, y	4	4469
Mean (SD)	72.0 (12.03)	70.5 (7.10)
Median	70.5	70.0
Range (Min, Max)	(60, 87)	(60, 96)
60 to 74	2 (50.0%)	3220 (72.1%)
75 to 84	1 (25.0%)	1069 (23.9%)
≥ 60 to 84	3 (75.0%)	4289 (96.0%)
≥ 85	1 (25.0%)	180 (4.0%)
Sex	4	4469
Female	4 (100.0%)	3040 (68.0%)
Male	0	1429 (32.0%)
Race	2	3703
African	0	175 (4.7%)
Asian	0	761 (20.6%)
Hispanic or Latino	0	7 (0.2%)
Indian	0	0
White	2 (100.0%)	2756 (74.4%)
Body mass index, kg/m^2^	4	4133
Mean (SD)	26.3 (5.17)	27.5 (5.75)
Median	25.9	26.7
Range (Min, Max)	(21, 33)	(14, 68)
Underweight < 18.5	0	420 (9.4%)
Normal 18.5 to ≤ 25	2 (50.0%)	1385 (31.1%)
Overweight 25 to ≤ 30	1 (25.0%)	1508 (33.9%)
Obese > 30	1 (25.0%)	1139 (25.6%)
Living status^a^	4	4469
At home	4 (100.0%)	4454 (99.7%)
Long-term care facility	0	3 (0.1%)
Assisted-living facility	0	3 (0.1%)
Other	0	9 (0.2%)
Travelled outside home country^b^	4	4469
Yes	0	310 (6.9%)
No	4 (100.0%)	3206 (71.7%)
Unknown	0	953 (21.3%)

Percentages are based on n with non-missing values

^a^Within 12 months prior to enrolment

^b^Within 6 months prior to enrolment

*FAS* full analysis set, *IED* invasive *Escherichia coli* disease

### Patients with IED

During 12 months of follow-up, four participants developed IED. All patients had a history of ≥ 1 IED risk–related medical condition and a history of UTI in the previous 10 years (Supplementary Table 4).

IED events were identified through self-report (*n* = 2), a GP report (*n* = 1), and a 4-month follow-up phone call by the LRT (*n* = 1) (Table 2). One IED event was reported outside the participating hospitals; diagnostic and treatment data were not available for this event. All IED events were *E. coli* bacteraemia and occurred in females with a history of UTI. *E. coli* was the only identified pathogen in all IED events. Clinical features, laboratory values, and *E. coli* culture results for IED events are summarised in Table 2. Additional tests performed in a diagnostic work-up of IED included electrocardiography, thoracic and chest x-ray, abdominal ultrasonography, computerised tomography of urinary tract, cystoscopy, blood culture, and urinalysis. None of the patients with IED died during the study period.

**Table 2:** Catchment process, clinical features, laboratory values, and *E. coli* culture results of 4 identified patients with IED

IED event catchment details	Signs and symptoms of UTI	Culture result	Signs and symptoms of IED	SIRS	qSOFA	SOFA^a^
IED event 1 (France)	NA		• Fever • General symptoms (malaise, fatigue, muscle pain, chills) • Abnormal MAP (mm Hg) • Laboratory values indicating bacterial infection and/or inflammation^b^	1	0	1
Catchment process: GP contacted LRT (GP was informed via hospital discharge letter)		Blood *E. coli* isolate				
IED event 2 (France)	NA		• Fever • Tachycardia (heart rate > 90 bpm) • General symptoms (malaise, fatigue, muscle pain, chills) • Nausea and/or vomiting • Abnormal MAP (mm Hg) • Laboratory values indicating bacterial infection and/or inflammation^b^	2	0	1
Catchment process: Participant informed EXPECT-2 PI that he also participates in EXPECT-1		Blood *E. coli* isolate				
IED event 3 (Italy)	• Dysuria • Flank pain		• Tachypnoea (respiratory rate > 20 breaths per minute) • Leukocytosis (WBC ≥ 12 × 10^9^/L) • Abnormal MAP (mm Hg) • Laboratory values indicating bacterial infection and/or inflammation^b^	2	1	1
Catchment process: Participant informed physician in hospital about participation in EXPECT-1		Blood *E. coli* isolate				
IED event 4 (Italy)	• Flank pain		• Leukocytosis (WBC ≥ 12 × 10^9^/L) • Laboratory values indicating bacterial infection and/or inflammation^b^	1	0	0
Catchment process: On the follow-up call on day 240, the LRT was informed by the participant that she had been admitted for IED		Blood *E. coli* isolate				

^a^When no prior SOFA score was available, the change in total score of ≥ 2 was calculated assuming the baseline SOFA score of 0

^b^Includes elevated C-reactive protein level and erythrocyte sedimentation rate

*bpm* beats per minute, *EXPECT-2* a second in the series of COMBACTE-NET studies to collect information about IED (ClinicalTrials.gov identifier: NCT04117113), *GP* general practitioner, *IED* invasive *Escherichia coli* disease, *LRT* local research team, *MAP* mean arterial pressure, *PI* principal investigator, *qSOFA* quick sequential organ failure assessment, *SIRS* systemic inflammatory response syndrome, *SOFA* sequential organ failure assessment, *UTI* urinary tract infection, *WBC* white blood cell

### Incidence of IED

The cumulative incidence of IED was 0.009% (95% CI, 0.024%–0.229%). The incidence rate of IED was 98.6 events per 100,000 person-years (95% CI, 25.6–248.4). Among participants with a history of UTI, the cumulative incidence of IED was 0.15% (95% CI, 0.041%–0.385%), and the incidence rate was 164.4 events per 100,000 person-years (95% CI, 43.8–420).

### Hospitalisations and medical encounters (FAS)

The number of hospitalisations reported during follow-up was 2,529 (56.6%) by the site and 2,177 (48.7%) by participants. The number of overlapping reports between the methods was 1,771 (39.6%) (Table 3). Of the total number of reported hospitalisations (*n* = 2,935), 86.2% would have been detected through site reports only. The median duration of hospitalisation was 1 day (Q1, 1.0; Q3, 15.0) when reported by the site (*n* = 2,230) and 1 day (Q1, 1.0; Q3, 2.0) when self-reported by participants (*n* = 1,661).

**Table 3:** Healthcare data reported by the site vs self-reported by participants and the number of overlapping reports between the two methods (FAS)

	Site report	Participant self-report	Overlapping reports
Full analysis set, N	4470	4470	
Number of participants hospitalised, *n* (%)	2529 (56.6)	2177 (48.7)	1771 (39.6)
Number of participants with any medical encounter, n (%)	3759 (84.1)	3480 (77.9)	3323 (74.3)
Type of medical encounter, per participant^a,b^	3759	3480	3323
General practitioner/family physician	2388 (63.5%)	2661 (76.5%)	2109
Hospital outpatient department	2189 (58.2%)	1774 (51.0%)	1371
Para-medical services	760 (20.2%)	840 (24.1%)	345
Emergency department	593 (15.8%)	618 (17.8%)	402
Hospital inpatient department	377 (10.0%)	417 (12.0%)	270
Home care	22 (0.6%)	11 (0.3%)	3
Intensive care unit	5 (0.1%)	5 (0.1%)	0
Number of participants with any procedure/intervention,^c^ n (%)	1966 (44.0%)	1976 (44.2%)	1493 (33.4%)
Diagnostic	1598 (81.3%)	1661 (84.1%)	1136
Interventional	661 (33.6%)	670 (33.9%)	419
	Site report	Participant self-report	Overlapping reports
Patients with an IED event, n (%)	4 (0.1)	4 (0.1)	-
Number of patients hospitalised, *n* (%)	4 (0.1)	4 (0.1)	-
Number of patients with any medical encounter, *n* (%)	4 (100.0)	4 (100.0)	-
Type of medical encounter, per patient^a,b^	4	4	-
Hospital inpatient department	4 (100.0%)	3 (75.0%)	-
Hospital outpatient department	4 (100.0%)	2 (50.0%)	-
General practitioner/family physician	2 (50.0%)	4 (100.0%)	-
Emergency department	1 (25.0%)	0	-
Para-medical services	0 (0)	1 (25.0%)	-
Number of patients with any procedure/intervention, *n* (%)	4 (100.0)	4 (100.0)	-
Diagnostic	4	4	-
Interventional	0	3	-

^a^Participants are counted only once for any given category but may appear in multiple categories

^b^Percentages in the specified group are calculated using the total number of participants with non-missing data in the denominator

^c^Number of participants excluding those with an IED event, *n* = 4,466

*FAS* full analysis set, *IED* invasive *Escherichia coli* disease

The number of participants with any medical encounter reported during follow-up was 3,759 (84.1%) by the site and 3,480 (77.9%) by participants. The number of overlapping reports between the methods was 3,323 (74.3%) (Table 3). Of the total number of reported medical encounters (*n* = 3,916), 96.0% would have been detected through site reports only. The total number and the number of overlapping reports between the site vs participants for different categories of medical encounters are summarised in Table 3. Excluding 4 patients with IED, the number of participants who underwent any procedure and/or intervention during a medical encounter was 1,966 (44.0%) when reported by the site and 1,976 (44.2%) when self-reported by participants. The number of overlapping reports between the methods was 1,493 (33.4%) (Table 3). Of the total number of reported procedures/interventions (*n* = 2,449), 80.3% would have been detected through site reports only.

### Hospitalisations and other medical encounters (patients with IED)

Hospitalisations, medical encounters, and diagnostic procedures/interventions performed during medical encounter were reported for all patients with IED by both methods (Table 3). The reported numbers of various types of medical encounters and interventional procedures differed depending on the method (Table 3).

### Treatment (patients with IED)

Antibiotics prescribed for the treatment of IED were intravenous (IV) meropenem followed by oral ciprofloxacin (*n* = 1); IV piperacillin–tazobactam followed by IV amoxicillin–clavulanate and oral amoxicillin–clavulanate (*n* = 1); and IV ceftriaxone followed by oral norfloxacin (*n* = 1).

### Geofencing

Between 23 March 2020 (the date when geofencing became available for consent) and 21 May 2021 (the date of the last visit), 334 US participants consented to EXPECT-1. Of those, 151 (45.2%) consented to the geofencing substudy, 60 (18.0%) downloaded the application on their personal cellular device, and 40 (12.0%) logged into it. Geofencing detected 72 hospital entries. All events were closed by site staff after patient follow-up to confirm the absence of infection.

## Discussion

This study prospectively identified four IED events through 12 months of follow-up in a population of 4,470 participants through the combined use of self-reports, GP reports, and phone-based follow-ups. The estimated incidence rate of IED was 98.6 events per 100,000 person-years. Among those with a history of UTI in the previous 10 years, the incidence rate increased to 164.4 events per 100,000 person-years.

The performance of a double-blind, placebo-controlled, randomised vaccine efficacy trial in enrolling older participants to detect an IED endpoint depends on utilising sufficiently large sample size as well as optimal detection of IED events through efficient tracking of hospitalisations and medical encounters. In this study, we estimated the percentage of reported healthcare events that would have been missed by utilising only one tracking method. For example, using only site reports would have missed approximately 4.0%–20.0% of hospitalisations, medical encounters, and procedures/interventions. As noted before, detection of IED events relied on a combined use of multiple tracking methods, suggesting an added benefit of combining site and participants’ reports in capturing healthcare data and IED events among older adults living in the community.

Certain discrepancies did arise from GP/PC reports compared to patient only reports. For example, hospital admissions were reported for all 4 participants by the GP/PC centers and by the participants, but the number of hospitalizations, duration, and outcome differed depending on whether it was reported by the GP/PC centers or by the participants. For 3 participants, a hospital admission was reported by the participant with IED as reason. For 1 participant, a hospital admission for cholecystectomy and hepatectomy was reported by the GP/PC center, not by the participant. The granularity of the data is insufficient to reach conclusions on the exact nature of the discrepancies. However, both the figures for the whole cohort, as the catchment method for the 4 patients with IED, suggest that a combination of different catchment methods is required to maximize the sensitivity of the study and as a consequence limit the number of required inclusions, and costs.

Detection of IED events in the community setting could be enhanced by digital technologies such as smartphone-based geofencing [11]. Although geofencing captured 72 hospital entries, but the study methods did not allow to confirm whether these were hospitalizations. To establish the utility of the geofencing method, future research should be designed to use a larger sample size and incorporate a control condition whereby hospitalisations would be ascertained through deployment of a reference standard (eg, GP reports). The current study identified barriers to implementing the geofencing approach in older adults. For example, difficulties in downloading the application on personal cellular devices, the lack of hands-on support from the site due to COVID-19, and participants’ privacy concerns likely contributed to the low application uptake. Future research could deploy strategies to mitigate these difficulties, for instance, by educating staff and participants that geofencing does not require continuous tracking of location, as the location is identified only after the electronic fence is crossed [11], and by assisting participants with installing the application during enrolment.

All participants who developed IED during follow-up were females with a history of UTI. Previous UTI treatment within a month before bacteraemia onset substantially increases the risk for urogenital tract–related bacteraemia [13]. By implication, *E. coli* infections localised within the urinary tract may progress to bacteraemia if sub-optimally treated. Data from epidemiological studies [13, 14] and a systematic review [7] identify the urinary tract as the most common source of *E. coli* bacteraemia. The link between UTI and IED, as indicated by the present data, might be more pronounced in older females, as both female sex and older age are well-documented risk factors for developing UTI [15, 16].

There are several limitations to this study. Generalisability of the findings on IED characterisation and treatment is limited due to detection of four IED events. One IED event was identified outside the participating hospitals, suggesting that restricting event detection within a hospital network might have limited the IED catchment efficiency. COVID-19 impacted enrolment, and less data were available to reach the study objectives. Finally, the reported rate of medical history of IED in the previous 10 years (7.2%) might have resulted from inaccurate assessment of IED, which was based on medical history review or the participant’s memory. The findings from EXPECT-1 provided insight into the feasibility of implementing an ongoing phase 3 trial of a vaccine against IED in community-dwelling older adults with a history of UTI in the past 2 years [9].

## Conclusion

This study identified four IED events in the population of community-dwelling older adults through the combined use of self-reports, GP reports, and phone-based follow-ups. Both site reports and participant self-reports appeared effective in tracking healthcare data among older adults living in the community. The addition of multitracking methods might enhance IED catchment.

### Supplementary Information

Below is the link to the electronic supplementary material.

Supplementary file1 (DOCX 57 KB)

## Data Availability

Although these data are not currently publicly available for sharing, requests for sharing can be sent to the Corresponding Author and will be evaluated on an individual basis.
