# Supplementary material for: Feasibility of tracking invasive Escherichia coli disease among older adults in a community setting: A prospective observational study
Source: Eur J Clin Microbiol Infect Dis. 2024 Jan 18;43(3):541–50. doi: 10.1007/s10096-023-04738-y (PMC10917840; doi:10.1007/s10096-023-04738-y)
Supplement: Supplementary file 1 — Supplementary file1 (DOCX 57 KB) [file 10096_2023_4738_MOESM1_ESM.docx]

**SUPPLEMENTARY INFORMATION**

**Feasibility of tracking invasive *Escherichia coli* disease among older adults in a community setting: A prospective observational study**

***European Journal of Clinical Microbiology & Infectious Diseases***

Joachim Doua^1^, Miquel Ekkelenkamp^2^, Theo Verheij^3^, Oscar Go^4^, Stephen Ruhmel^5^, Kimberly Leathers^6*^, Bart Spiessens^1^, Sanne van Rooij^3^, Vance G. Fowler Jr.^7,8^, Jeroen Geurtsen^9^, Rowena Dolor^6^, Michal Sarnecki^10^, Ranee Chatterjee^6^, Jan Poolman^9^, Marc Bonten^3^ on behalf of COMBACTE-NET consortium/EXPECT study group

**Affiliations:** ^1^Janssen Research & Development, Beerse, Belgium; ^2^Department of Medical Microbiology, University Medical Center Utrecht, Utrecht University, Utrecht, the Netherlands; ^3^Julius Center for Health Sciences and Primary Care, University Medical Center, Utrecht University, Utrecht, the Netherlands; ^4^Janssen Research & Development, Raritan, NJ, USA; ^5^Janssen Research & Development, Titusville, NJ, USA; ^6^Duke University School of Medicine, Durham, NC, USA; ^7^Department of Medicine, Duke University Medical Center Durham, NC, USA; ^8^Duke Clinical Research Institute, Durham, NC, USA; ^9^Bacterial Vaccines Discovery and Early Development, Janssen Vaccines & Prevention B.V, Leiden, the Netherlands; ^10^Janssen Vaccines, Branch of Cilag GmbH International, Bern, Switzerland *Current affiliation: IQVIA, Research Triangle Park, NC, USA

**Corresponding author**: Michal Sarnecki, Janssen Vaccines, Branch of Cilag GmbH International, Bern, Switzerland; +41 (0)31 561 10 71, email: [msarneck@its.jnj.com](mailto:msarneck@its.jnj.com).

**EXPECT-1 principal investigators**

| **Principal investigator** | **Organization/region** |
| --- | --- |
| McElhaney, Janet | Northern Ontario School of Medicine |
| Römer, Katja | Gemeinschaftspraxis |
| Wyen, Christoph | Praxis Ebertplatz |
| Biehl, Lena | UKK Uniklinik Köln |
| Hunsdorfer, Frauke | Hausärztliche internistische Praxis |
| Martínez Gacio, Eva María | Centro de Salud D. Manuel Marroco Quevedo de Cantillana |
| Villena Machuca, María Luisa | Centro de Salud de Alcalá del Río |
| Fernández Castillo, María Nieves | Centro de Salud de Guillena |
| González Martínez, Carmen Macarena | Centro de Salud de La Algaba |
| Masía Perpiña, Óscar Rafael | Centro de Salud Los Carteros San José de la Rinconada |
| Baena Martín, Fernando | Centro de Salud Santa María de Gracia de Carmona |
| Berrocal Peña, Juan Manuel | Centro de Salud Virgen de Setefilla de Lora del Río |
| Rodríguez-Baño, Jesús | Hospital Universitario Virgen Macarena |
| Moesch, Cyril | USSEL |
| Bussy, Marie | Bourganeuf |
| Guillaumot, Hervé | Bourganeuf |
| Buchon, Daniel | Bugeat |
| Grenaille, Timothée | Bugeat |
| Faure, Christian | Limoges – Cabinet Medical du midi – FAURE |
| Pfender, Elodie | Centre Hospitalier Universitaire de Limoges |
| Prioux, Catherine | Faux-la-Montagne |
| Larque, Fabrice | Le Palais-sur-Vienne |
| Atgie, Florence | Limoges |
| Audoin-Picot, Carole | Limoges |
| Baldy, Bruno | Limoges |
| Bouyoux, Gaëlle | Limoges |
| Dumoitier, Nathalie | Limoges |
| Fleurat, François-Louis | Limoges |
| Godard, Severine | Limoges |
| Gory, Stephanie | Limoges – Cabinet Médical du midi |
| Jacquet, Quentin | Limoges |
| Lauchet, Nadege | Limoges |
| Martial Marzolff, Yannick | Limoges |
| Nouhaud, Serge | Limoges |
| Riviere, Olivier | Limoges |
| Beylie, Pierre | Lubersac |
| Jacob, Pascale | Lubersac |
| Delage, Pierre-David | Nexon |
| Delage-Pommier, Magali | Nexon |
| Salin-LeBlanc, Laure | Veyrac |
| Baclet, Marie-Claire | Oradour-sur-Vayres |
| Lagrace, Olivier | Saint-Just-le-Martel |
| Pautout, Marie-Paule | Saint-Just-le-Martel |
| DeBlaere-Bastos, Isabelle | Saint-Léonard-de-Noblat |
| Rudelle, Karen | Saint-Yrieix-la-Perche |
| Collet, Nadège | Saint-Yrieix-la-Perche |
| Seve, Léa | Seilhac |
| Binnian, Ian | Eynsham Medical Group |
| Wooding, Nick | South Oxford Health Centre |
| Tacconelli, Evelina | Azienda Ospedaliera Universitaria Integrata di Verona |
| Izumi, Yasumori | Nagasaki Medical Center |
| Abe, Akihiko | Okitama Public General Hospital |
| Koga, Shigehiko | Kurogi Clinic |
| Minamino, Takeshi | Minamino Clinic |
| Miyashita, Taiichiro | Miyashita Rheumatology Clinic |
| Noguchi, Akihiko | Noguchi Internal Medicine Child Clinic |
| Oka, Hiroyuki | Oka Clinic |
| Onmura, Yoshiki | Okitama Nagai Public Hospital |
| Sadamatsu, Toshihiro | Sadamatsu Hospital |
| Ishida, Kenji | Sakura Clinic |
| Tanaka, Kyoe | Tanaka Cardiology Clinic |
| Tomonaga, Akimitsu | Tomonaga Clinic |
| Yamashita, Naohiro | Yamashita Clinic |
| Terai, Yuji | Terai Clinic |
| Thaden, Joshua | PCRC Duke University |
| Chatterjee, Ranee | PCRC Duke University |
| Vallejo, Christine | Centre Hospitalier Universitaire de Limoges |
| Inage, Minoru | Okitama Public General Hospital |
| Walker, Sarah | Oxford Radcliffe Hospitals NHS Trust John Radcliffe Hospital |

**Supplementary Table 1** Study exclusion criteria

| **Exclusion** | **Further detail** |
| --- | --- |
| **Serious chronic disorder** | - Severe chronic obstructive pulmonary disease - Clinically significant congestive heart failure - End-stage renal disease with or without dialysis - Clinically unstable cardiac disease - Alzheimer disease - Any condition for which, in the opinion of the investigator, participation would not be in the best interest of the participant (eg, it would compromise well-being) or any condition that could prevent, limit, or confound the protocol-specified assessments |
| **History of malignancy within 5 years before screening** | - Exceptions: squamous and basal cell carcinomas of the skin, carcinoma in situ of the cervix, or malignancy that is considered cured with minimal risk of recurrence |
| **Major psychiatric illness and/or substance abuse or substance use disorder** | - Psychiatric illness which, in the opinion of the investigator, would compromise the participant’s safety and/or compliance with study procedures |
| **Likelihood of non-adherence** | - Participant who, in the opinion of the investigators, is unlikely to adhere to the requirements of the study, or is unlikely to complete the full course of the study |

**Supplementary Table 2** A list of medical conditions considered to carry increased risk for developing IED

| **IED risk–related medical history** |
| --- |
| UTI |
| Uncomplicated UTI |
| Complicated UTI |
| Recurrent UTI |
| Cardiovascular disease |
| Gastrointestinal disease |
| Cerebrovascular accident (stroke) |
| Diabetes mellitus |
| Urolithiasis |
| Urological intervention |
| Other conditions with increased risk for IED |

*IED* invasive *Escherichia coli* disease, *UTI* urinary tract infection

**Supplementary Table 3** *International Classification of Diseases* codes used to identify invasive *Escherichia coli* disease

| *ICD* code | Group | Meaning | To be used in conjunction with laboratory codes^a^ |
| --- | --- | --- | --- |
| B96 |  | Other bacterial agents as the cause of diseases classified to other chapters | 1 to 3 |
| B96.2 | Bacterial agents | *E. coli* as the cause of diseases classified elsewhere | NA |
| B96.20 |  | Unspecified *E. coli* as the cause of diseases classified elsewhere | NA |
| B96.29 |  | Other *E. coli* as the cause of diseases classified elsewhere | NA |
| A41 |  | Other sepsis | 1 to 3 |
| A41.5 |  | Sepsis due to other gram-negative organisms | 1 to 3 |
| A41.50 |  | Gram-negative sepsis, unspecified | 1 to 3 |
| A41.51 |  | Sepsis due to *E. coli* | NA |
| A41.8 |  | Other specified sepsis | 1 to 3 |
| A41.89 | Infectious | Other specified sepsis | 1 to 3 |
| A41.9 | diseases | Sepsis, unspecified organism | 1 to 3 |
| A48 |  | Other bacterial diseases, not elsewhere classified | 1 to 3 |
| A48.8 |  | Other specified bacterial diseases | 1 to 3 |
| A49 |  | Bacterial infection of unspecified site | 1 to 3 |
| A49.8 |  | Other bacterial infection of unspecified site | 1 to 3 |
| A49.9 |  | Bacterial infection, unspecified | 1 to 3 |
| D73.3 | Diseases of blood and blood-forming organs | Abscess of spleen | 1 to 3 |
| G00 |  | Bacterial meningitis, not elsewhere classified | 1 to 3 |
| G00.8 |  | Other bacterial meningitis | 1 to 3 |
| G00.9 | Diseases of the | Bacterial meningitis, unspecified | 1 to 3 |
| G01 | nervous system | Meningitis in bacterial diseases classified elsewhere | 1 to 3 |
| G02 |  | Meningitis in other infectious and parasitic diseases classified elsewhere | 1 to 3 |
| G03 |  | Meningitis due to other and unspecified causes | 1 to 3 |
| G03.9 |  | Meningitis, unspecified | 1 to 3 |
| G06^⁑^ |  | Intracranial and intraspinal abscess and granuloma | 1 to 3 |
| I30.1 |  | Infective pericarditis | 1 to 3 |
| I33^⁑^ | Diseases of the | Acute and subacute infective endocarditis | 1 to 3 |
| I38 | circulatory system | Endocarditis, valve unspecified | 1 to 3 |
| I39 |  | Endocarditis and heart valve disorders in diseases classified elsewhere | 1 to 3 |
| I77.6 |  | Arteritis, unspecified | 1 to 3 |
| J15 |  | Bacterial pneumonia, not elsewhere classified | 1 to 3 |
| J15.5 |  | Pneumonia due to *E. coli* | NA |
| J15.9 |  | Unspecified bacterial pneumonia | 1 to 3 |
| J16.8 |  | Pneumonia due to other specified infectious organisms | 1 to 3 |
| J17 |  | Pneumonia in diseases classified elsewhere | 1 to 3 |
| J18  J18.0 | Diseases of the respiratory system | Pneumonia, unspecified organism  Bronchopneumonia, unspecified organism | 1 to 3 |
| J18.1 |  | Lobar pneumonia, unspecified organism | 1 to 3 |
| J18.2 |  | Hypostatic pneumonia, unspecified organism | 1 to 3 |
| J18.8 |  | Other pneumonia, unspecified organism | 1 to 3 |
| J18.9 |  | Pneumonia, unspecified organism | 1 to 3 |
| J85^⁑^ |  | Abscess of lung and mediastinum | 1 to 3 |
| J86^⁑^ |  | Pyothorax | 1 to 3 |
| J98.51 |  | Mediastinitis | 1 to 3 |
| K61^⁑^ |  | Abscess of anal and rectal regions | 1 to 3 |
| K63.0 |  | Abscess of intestine | 1 to 3 |
| K65 |  | Peritonitis | 1 to 3 |
| K65.0 |  | Generalized (acute) peritonitis | 1 to 3 |
| K65.1 |  | Peritoneal abscess | 1 to 3 |
| K65.2 |  | Spontaneous bacterial peritonitis | 1 to 3 |
| K65.8 |  | Other peritonitis | 1 to 3 |
| K65.9 |  | Peritonitis, unspecified | 1 to 3 |
| K67.8 | Diseases of the digestive system | Disorders of peritoneum in infectious diseases classified elsewhere | 1 to 3 |
| K68.1^⁑^ |  | Retroperitoneal abscess | 1 to 3 |
| K75.0 |  | Abscess of liver | 1 to 3 |
| K83.0 |  | Cholangitis | 1 to 3 |
| K85.8.^⁑^ |  | Other acute pancreatitis | 1 to 3 |
| K85.9 |  | Acute pancreatitis, unspecified | 1 to 3 |
| M00.8^⁑^ |  | Arthritis and polyarthritis due to other bacteria | 1 to 3 |
| M00.9 |  | Pyogenic arthritis, unspecified | 1 to 3 |
| M01^⁑^ | Diseases of the musculoskeletal  system and  connective tissue | Direct infections of joint in infectious and parasitic diseases classified elsewhere | 1 to 3 |
| M46.3 |  | Infection of intervertebral disc (pyogenic) | 1 to 3 |
| M60.0 |  | Infective myositis | 1 to 3 |
| M71.0^⁑^ |  | Abscess of bursa | 1 to 3 |
| M71.1^⁑^ |  | Other infective bursitis | 1 to 3 |
| M72.6 |  | Necrotising fasciitis | 1 to 3 |
| M86^⁑^ |  | Osteomyelitis | 1 to 3 |
| N10 |  | Acute pyelonephritis | 1 to 3 |
| N11^⁑^ |  | Chronic tubulointerstitial nephritis | 1 to 3 |
| N15.1  N29 |  | Renal and perinephric abscess  Other disorders of kidney and ureter in diseases classified elsewhere | 1 to 3  1 to 3 |
| N30 |  | Cystitis | 1 to 3^b^ |
| N30.0^⁑^ |  | Acute cystitis | 1 to 3^b^ |
| N30.1^⁑^ |  | Interstitial cystitis (chronic) | 1 to 3^b^ |
| N30.2^⁑^ |  | Other chronic cystitis | 1 to 3^b^ |
| N30.3^⁑^ |  | Trigonitis | 1 to 3^b^ |
| N30.5^⁑^ | Diseases of the | Other cystitis | 1 to 3^b^ |
| N30.9^⁑^ | genitourinary | Cystitis, unspecified | 1 to 3^b^ |
| N34 ^⁑^ | system | Urethritis and urethral syndrome | 1 to 3^b^ |
| N39 |  | Urinary tract infection, site not specified | 1 to 3^b^ |
| N41.0 |  | Acute prostatitis | 1 to 3^b^ |
| N41.1 |  | Chronic prostatitis | 1 to 3^b^ |
| N41.2 |  | Abscess of prostate | 1 to 3^b^ |
| N41.3 |  | Prostatocystitis | 1 to 3^b^ |
| N43.1 |  | Infected hydrocele | 1 to 3 |
| N45^⁑^ |  | Orchitis and epididymitis | 1 to 3 |
| N70^⁑^ |  | Salpingitis and oophoritis | 1 to 3 |
| N76.4 |  | Abscess of vulva | 1 to 3 |
| O75.3 |  | Sepsis during labour | 1 to 3 |
| O03.37 |  | Sepsis following incomplete spontaneous abortion | 1 to 3 |
| O03.87 |  | Sepsis following complete or unspecified spontaneous abortion | 1 to 3 |
| O04.87 |  | Sepsis following (induced) termination of pregnancy | 1 to 3 |
| O07.37 | Pregnancy related | Sepsis following failed attempted termination of pregnancy | 1 to 3 |
| O08.82 |  | Sepsis following ectopic and molar pregnancy | 1 to 3 |
| O85 |  | Puerperal sepsis | 1 to 3 |
| O86^⁑^ |  | Other puerperal infection | 1 to 3 |
| O91.1^⁑^ |  | Abscess of breast associated with pregnancy, the puerperium, and lactation | 1 to 3 |
| R09.1 |  | Pleurisy | 1 to 3 |
| R50 |  | Fever of other and unknown origin | 1 to 3 |
| R50.9 |  | Fever, unspecified | 1 to 3 |
| R65 | Symptoms, signs, and laboratory findings not elsewhere classified | Symptoms and signs specifically associated with systemic inflammation and infection | 1 to 3 |
| R65.10 |  | SIRS of non-infectious origin without acute organ dysfunction | 1 to 3 |
| R65.11 |  | SIRS of non-infectious origin with acute organ dysfunction | 1 to 3 |
| R65.2 |  | Severe sepsis | 1 to 3 |
| R65.20 |  | Severe sepsis without septic shock | 1 to 3 |
| R65.2 |  | Severe sepsis with septic shock | 1 to 3 |
| R78.81 |  | Bacteraemia | 1 to 3 |
| T80.211 | Complications of | Bloodstream infection due to central venous catheter | 1 to 3 |
| T80.219 | surgical or | Unspecified infection due to central venous catheter | 1 to 3 |
| T80.22 | medical care not elsewhere classified | Acute infection following infusion, transfusion, and therapeutic injection | 1 to 3 |
| T81.3 |  | Disruption of wound, not elsewhere classified | 1 to 3 |
| T81.4 |  | Infection following a procedure | 1 to 3 |
| T81.89 |  | Other complications of procedures, not elsewhere classified | 1 to 3 |
| T81.9 |  | Unspecified complication of procedure | 1 to 3 |
| D78.89 |  | Other post-procedural complications of the spleen | 1 to 3 |
| G97.82 | Potential surgical site infections | Other post-procedural complications and disorders of nervous system | 1 to 3 |
| I97.89 |  | Other post-procedural complications and disorders of the circulatory system, not elsewhere classified | 1 to 3 |
| J95.89 |  | Other post-procedural complications and disorders of respiratory system, not elsewhere classified | 1 to 3 |
| K91.89 |  | Other post-procedural complications and disorders of digestive system | 1 to 3 |
| L76.82 |  | Other post-procedural complications of skin and subcutaneous tissue | 1 to 3 |
| N99.89 |  | Other post-procedural complications and disorders of genitourinary system | 1 to 3 |

^a^As an alternative to the *ICD* codes, sites may combine the clinical codes with information from the laboratory showing a positive *E. coli* culture

^b^Cultured from urine

^⁑^Plus all other lower level codes

*ICD International Classification of Diseases NA* not applicable, *SIRS* systemic inflammatory response syndrome

**Supplementary Table 4** IED risk–related medical history

|  | **Non-IED** | **IED** | **Total** |
| --- | --- | --- | --- |
| FAS, n | 4466 | 4 | 4470 |
| Participants with a history of any of the following diseases,^a^ n (%) | - | 4 (100.0) | - |
| UTI | - | 4 (100.0) | - |
| Recurrent UTI | - | 2 (50.0) | - |
| Cardiovascular disease | - | 2 (50.0) | - |
| Gastrointestinal disease | - | 2 (50.0) | - |
| Cerebrovascular accident (stroke) | - | 1 (25.0) | - |
| Diabetes mellitus | - | 1 (25.0) | - |
| Urolithiasis | - | 1 (25.0) | - |
| Urological intervention | - | 1 (25.0) | - |
| Other conditions with increased risk for IED, n (%) | - | 1 (25.0) | - |
| Uncomplicated UTI, n (%) | - | 1 (25.0) | - |
| Complicated UTI, n (%) | - | 1 (25.0) | - |
| History of UTI in the previous 10 years, n (%) |  |  |  |
| n | 4465 | 4 | 4469 |
| No UTI | 1812 (40.6) | - | 1812 (40.5) |
| < 2 years prior to enrolment | 492 (11.0) | - | 492 (11.0) |
| ≥2 years | 2098 (47.0) | 4 (100.0) | 2102 (47.0) |
| Unknown date | 63 (1.4) | - | 63 (1.4) |
| History of IED in the previous 10 years, n (%) |  |  |  |
| n | 4465 | 4 | 4469 |
| No IED | 3536 (79.2) | 2 (50.0) | 3538 (79.2) |
| With IED | 323 (7.2) | 1 (25.0) | 324 (7.2) |
| Unknown | 606 (13.6) | 1 (25.0) | 607 (13.6) |

^a^IED risk–related medical history data were only collected in participants with IED

*FAS* full analysis set, *IED* invasive *Escherichia coli* disease, *UTI* urinary tract infection

**Supplementary Table 5** Demographic and baseline characteristics in participants with and without a history of urinary tract infection in the previous 10 years (FAS)

|  | **With UTI History** | **No History of UTI** |
| --- | --- | --- |
| Analysis set: FAS | 2657 | 1812 |
| Age, y | 2657 | 1812 |
| Mean (SD) | 70.9 (7.27) | 69.9 (6.81) |
| Median | 70.0 | 69.0 |
| Range (Min, Max) | (60, 96) | (60, 93) |
| 60 to 74 | 1848 (69.6%) | 1372 (75.7%) |
| 75 to 84 | 685 (25.8%) | 384 (21.2%) |
| ≥60 to 84 | 2533 (95.3%) | 1756 (96.9%) |
| ≥85 | 124 (4.7%) | 56 (3.1%) |
| Sex | 2657 | 1812 |
| Female | 2059 (77.5%) | 981 (54.1%) |
| Male | 598 (22.5%) | 831 (45.9%) |
| Race | 2242 | 1461 |
| African | 98 (4.4%) | 77 (5.3%) |
| Asian | 430 (19.2%) | 331 (22.7%) |
| Hispanic or Latino | 5 (0.2%) | 2 (0.1%) |
| Indian | 0 | 0 |
| White | 1706 (76.1%) | 1050 (71.9%) |
| Body mass index, kg/m^2^ | 2461 | 1672 |
| Mean (SD) | 27.5 (5.93) | 27.6 (5.49) |
| Median | 26.6 | 26.9 |
| Range (Min, Max) | (15, 68) | (14, 62) |
| Underweight <18.5 | 249 (9.4%) | 170 (9.4%) |
| Normal 18.5 to ≤25 | 828 (31.3%) | 557 (30.8%) |
| Overweight 25 to ≤30 | 897 (33.9%) | 611 (33.8%) |
| Obese >30 | 670 (25.3%) | 469 (26.0%) |
| Living status^a^ | 2657 | 1812 |
| At home | 2645 (99.5%) | 1809 (99.8%) |
| Long-term care facility | 3 (0.1%) | 0 |
| Assisted-living facility | 2 (0.1%) | 1 (0.1%) |
| Other | 7 (0.3%) | 2 (0.1%) |
| Travelled outside home country^b^ | 2657 | 1812 |
| Yes | 177 (6.7%) | 133 (7.3%) |
| No | 2002 (75.3%) | 1204 (66.4%) |
| Unknown | 478 (18.0%) | 475 (26.2%) |
| ^a^Within 12 months prior to enrolment  ^b^Within 6 months prior to enrolment  *FAS* full analysis set, *UTI* urinary tract infection | | |
